# Relationship Between Socioeconomic Status and Job Accommodation for Workers with Health Problems in Japan during the COVID-19 Pandemic

**DOI:** 10.1016/j.shaw.2025.04.002

**Published:** 2025-04-10

**Authors:** Yu Igarashi, Seiichiro Tateishi, Arisa Harada, Kosuke Mafune, Mayumi Tsuji, Akira Ogami, Tomohisa Nagata, Ryutaro Matsugaki, Yoshihisa Fujino, for the CORoNaWork project

**Affiliations:** 1 Disaster Occupational Health Center, Institute of Industrial Ecological Sciences, University of Occupational and Environmental Health, Japan; 2 Department of Occupational Medicine, School of Medicine, University of Occupational and Environmental Health, Japan; 3 Department of Mental Health, Institute of Industrial Ecological Sciences, University of Occupational and Environmental Health, Japan; 4 Department of Environmental Health, School of Medicine, University of Occupational and Environmental Health, Japan; 5 Department of Work Systems and Health, Institute of Industrial Ecological Sciences, University of Occupational and Environmental Health, Japan; 6 Department of Occupational Health Practice and Management, Institute of Industrial Ecological Sciences, University of Occupational and Environmental Health, Japan; 7 Department of Preventive Medicine and Community Health, School of Medicine, University of Occupational and Environmental Health, Japan; 8 Department of Environmental Epidemiology, Institute of Industrial Ecological Sciences, University of Occupational and Environmental Health, Japan

**Keywords:** Covid-19, Japan, Socioeconomic status, Job accommodation, Worker

## Abstract

**Background:**

During the COVID-19 pandemic, many health-impaired workers of vulnerable socioeconomic status (SES) suffered employment and health problems. This study investigated the relationship between workers with health problems in vulnerable SES and job accommodation.

**Methods:**

This cross-sectional internet monitoring study was conducted among 33,302 Japanese workers from December 22 to 26, 2020. Of the total survey participants, 6,309 who reported that they needed accommodations from their companies were included in the analysis. Using a multilevel logistic regression model, we examined the relationship between their SES and the lack of job accommodations from their companies, and age-sex-adjusted and multivariate-adjusted odds ratios (ORs) were estimated.

**Results:**

Multivariate analysis revealed that the OR for the lack of accommodations from companies was significantly higher for non-desk jobs than desk jobs (OR=1.15, 95% CI: 1.04–1.28, p=0.01). The ORs also differed based on household incomes and number of employees. ORs were significantly higher for those with an equivalent household income of less than 2.9 million yen compared with 9 million yen or more (OR=1.66, 95% CI: 1.39–1.97, p<0.01). Comparing with firms with 1,000 or more employees, the ORs were significantly higher for those with fewer than 30 employees (OR=1.21, 95% CI: 1.05–1.40, p<0.01).

**Conclusions:**

We found a relationship between SES and the lack of job accommodation for workers with health problems. The lack of job accommodation may further worsen SES and health conditions, reinforcing the importance of accommodating the needs of vulnerable SES workers.

## 1 Introduction

In recent years, the number of workers who can work even though they have health problems has been increasing, and the workstyle of workers with health problems has become a significant issue in occupational health [1]. The causes of this increase include the increasing number of older workers with chronic diseases in the aging society and the raising of the retirement age [2], improvements in the treatment of vascular diseases, cancer, and other diseases due to advances in medical technology [[3], [4], [5]], and the increasing employment of disabled workers [6,7]. Workers at risk regarding issues of occupational health and safety [8], labor productivity [9], and workplace retention rates [10] have become company management and operational concerns. In parallel, for workers with health problems, continuing to work is essential not only for economic reasons but also for social participation, self-efficacy, and quality of life [11,12].

From a labor market perspective, companies are expected to support people with health problems who continue to work. Many such workers can continue working if they receive appropriate support [[13], [14], [15]]. However, it has been reported that workers with health problems are unfairly excluded from the labor market [16]. Such exclusion can lead to long-term unemployment, poverty, and social security burdens [17]. Studies have revealed that job accommodations for workers is associated with low turnover in those with mental illness [14], continued employment after returning to work in those with cancer [18,19], and longer duration of employment in those with disabilities [13]. These accommodations are not general features of jobs with inherent flexibility but targeted measures addressing specific health-related needs. This indicates the need to support employees as an aspect of corporate social responsibility.

In this study, “job accommodation” specifically refers to adjustments made to the work process or environment to enable individuals with disabilities or health problems to work effectively. This is distinct from general workplace benefits provided to all workers. In Japan, a government initiative is underway to establish a support system to enable workers with health problems to continue treatment and work [6,20,21]. Under this initiative, called “Ryoritsu shien,” companies will provide appropriate accommodations in their work system to workers with health problems to enable them to balance work and medical treatment. Government campaigns, guidelines, and revisions will be introduced into the medical insurance system under which the attending physician will support the patient’s work. Accommodations provided by companies could include adjustment of employee work hours or content to take account of their physical condition and symptoms, and thereby enable them to continue working during treatment. For example, a company might modify job descriptions for workers with mental illnesses according to their symptoms, improve communication channels, provide flexible work schedules for workers with cancer who are undergoing treatment, and facilitate breaks for those who are easily fatigued. Similar efforts are being made in the United States (U.S.) and the United Kingdom (U.K.). In the U.S., a Job Accommodation Network Association disseminates information about job accommodation for various diseases [22]. In the UK, general practitioners play a central role by providing medical advice on working to people with health problems in the form of “Fit notes,” and companies provide accommodation based on these Fit notes [23].

However, not all workers receive accommodation from their companies. Previous studies have reported that vulnerable socioeconomic status (SES) workers with cancer or psychiatric disorders are less likely to receive job accommodations [19,24,25]. Furthermore, 30% of workers with mental disabilities in Canada [24] and 40% of cancer survivors in France [19] do not receive accommodations. Zwerling et al. (2003) found that males with mental illness are less likely to receive accommodations than college graduates and older, full-time, or self-employed workers in the US [25]. Low-SES workers are trapped in a vicious cycle of working under poor health conditions, which further lowers their SES. People of vulnerable SES with health problems in particular are unable to receive appropriate treatment because of a lack of access to sick leave programs; instead, they work with employment insecurity, which reduces productivity and results in resignations or retirement [26,27].

During the COVID-19 pandemic, vulnerable SES workers with health problems suffered employment and health issues. The employment environment deteriorated due to low economic activity, which resulted in low employment rates and wages, especially pronounced for workers with health problems [28,29]. Furthermore, vulnerable SES workers had higher COVID-19 infection and mortality rates than high-SES workers, due to the formers’ inability to telework and unsanitary working conditions associated with greater risks of infection, comorbidities, and lack of access to medical care [30,31]. Their health problems were exacerbated because of interruptions in their treatments and worsened by anxiety arising from infection fears [32,33]. Hence, more job accommodation is required for vulnerable SES workers with health problems during the pandemic.

To date, few studies have investigated the relationship between SES and accommodations from companies for workers with health problems, especially in Japan. Therefore, this study hypothesized that “workers with health problems and vulnerable SES do not receive job accommodation from their companies,” and investigated this relationship.

## 2 Materials and methods

### 2.1 Study design and participants

The Collaborative Online Research on Novel-coronavirus and Work (CORONaWork) project is an Internet-based cross-sectional monitoring survey that was conducted from December 22 to 26, 2020. The survey was conducted using an internet research company with 4.7 million registered individuals. The project examined the relationship between the working environment and workers’ health status during the COVID-19 pandemic [34]. The survey targeted workers aged 20–65 years and residing in Japan. A survey request was sent to 6,05,381 individuals. From the 55,045 who responded, we sampled equally for sex, age, and occupation for each of the five regions of residence classified by infection status. Then, 27,036 valid responses were collected after excluding respondents with extremely short response times (<6 minutes), less than 140 cm in height, less than 30 kg in weight, contradictory responses to multiple identical questions, and answers to questions designed to identify fake responses. In this study, job accommodation refers to specific adjustments or modifications in the work process or environment provided by employers and supervisors to enable workers with health problems or disabilities to perform their jobs effectively. This definition aligns with that provided by the American Job Accommodation Network. Therefore, we excluded 2,607 respondents from the 27,036 who were working in small office/home office settings or self-employed as they were not eligible to receive accommodations from their companies. Furthermore, since job accommodations are intended for individuals in poor health who require support, 6,309 respondents were analyzed, while 18,120 respondents who indicated that they did not require any special accommodations were excluded.

### 2.2 Evaluation of accommodations by companies for workers with health problems

We asked, “*Do you need any job accommodations from your company to continue working with your current health condition?*” to determine the workers’ need. Participants responded by selecting “*Not necessary*,” “*Yes, but I am not receiving any accommodations*,” and “*Yes, I am receiving accommodations*.” Those who answered “*Not necessary*” were excluded. Furthermore, those who answered, “*Yes, but I am not receiving any accommodations*” were defined as “in poor health and without receiving accommodation” and those who answered, “*Yes, I am receiving accommodations*” were defined as “in poor health and receiving accommodation.”

#### 2.2.1 Assessment of SES

We assessed marital status, equivalent household income, educational background, occupation, number of employees in the company, employment status, and subjective economic status. *Marital status* was categorized into three groups: married, never married, and divorced or widowed. *Equivalent household income* was classified into four groups: less than 4 million Japanese Yen, 4–5.99 million Yen, 6–8.99 million Yen, and 9 million Yen or more. *Educational background* was classified into three groups: junior high school graduates, high school graduates, and graduates of vocational schools and universities. *Occupations* were categorized into two groups: mainly desk work and non-desk work. *Number of employees* was classified into five groups: below 29, 30–99, 100–499, 500–999, and 10,000 or more employees. *Employment status was* categorized into two groups: regular worker and non-regular worker. *Subjective economic status* was classified into five groups: very comfortable, comfortable, fairly difficult, slightly difficult, and very difficult. Drinking habits were classified by the frequency of drinking alcohol, smoking habits by smoking status, and exercise habits by the frequency of exercising for more than 30 minutes. Comorbidities were assessed by the presence or absence of illnesses requiring regular or hospital visits or treatment.

### 2.3 Other covariates

We assessed whether participants felt that they received support from their supervisors and coworkers. Support from supervisors was evaluated by summing the answers to three questions: “*My supervisor thinks of what is in my best interests*,” “*My supervisor listens to what I say,*” and “*My supervisor helps me get the job done*.” The level of support from supervisors was divided into four levels: *extreme, very much, somewhat,* and *not at all.*

Support from coworkers was evaluated by the sum of the answers to three questions: “*The people I work with take a personal interest in me,*” “*The people I work with are approachable,*” and “*The people I work with help me get the job done.*” The level of support from coworkers was divided into four groups: *extreme, very much, somewhat,* and *not at all.*

### 2.4 Statistical analysis

We examined the relationship between each variable and the lack of job accommodation using a multivariate logistic regression model, with receiving accommodation as the dependent variable and SES as the independent variable. Age- and sex-adjusted and multivariate-adjusted odds ratios (ORs) were estimated. Multivariate models (model 2) were adjusted for sex, age, equivalent household income, and education. The multivariate model (model 3) was adjusted for alcohol, smoking, exercise habits, and comorbidities in addition to model 2. Four adjustment variables were selected to avoid multicollinearity as other variables may be correlated with each other. Categorical variables were converted to dummy variables for multivariate analysis. The basic characteristics of the study participants were compared using *t* tests for continuous variables and chi-square tests for categorical variables. The *p* values less than 0.05 were considered statistically significant. All analyses were performed using R ver. 4.2.2 (R Foundation for Statistical Computing, Vienna, Austria) [35], and the Strengthening the Reporting of Observational Studies in Epidemiology guideline for cross-sectional studies was used to prepare this report [36].

### 2.5 Ethical consideration

This study was approved by the ethics committee of the University of Occupational and Environmental Health, Japan (Reference No. R2-079 and R3-006).

## 3 Results

Table 1 presents the participant characteristics. Of the 6,309 participants analyzed in this study, 2,310 were “*in poor health and receiving accommodation,*” and 3,999 were “*in poor health and without receiving accommodation.*”

**Table 1:** Basic characteristics of the study participants

	Sick workers with accommodation n = 2,310	Sick workers without accommodation n = 3,999	*p* value
Age (median)	46 (36, 53)	47 (38, 54)	<0.01
Sex male	1,082 (47%)	1,985 (50%)	0.03
Marital status, married	1,238 (54%)	2,034 (51%)	0.04
Job type			<0.01
Mainly desk work	1,159 (50%)	1,831 (46%)	
Mainly non-desk work	1,151 (50%)	2,168 (54%)	
Annual equivalent household income (JPY)			<0.01
-2,990,000	500 (22%)	695 (17%)	
3,000,000–4,990,000	437 (19%)	593 (15%)	
5,000,000–6,990,000	484 (21%)	851 (21%)	
7,000,000–8,990,000	549 (24%)	1,090 (27%)	
9,000,000–	340 (15%)	770 (19%)	
Education			<0.01
Junior high school	1,739 (75%)	2,867 (72%)	
High school	545 (24%)	1,061 (27%)	
Vocational school/college, university, graduate school	26 (1%)	71 (2%)	
Number of employees			<0.01
1000–	729 (32%)	1028 (26%)	
500–999	208 (9%)	354 (9%)	
100∼499	440 (19%)	892 (22%)	
30∼99	379 (16%)	697 (17%)	
-29	554 (24%)	1,028 (26%)	
Employment status			0.06
Regular worker	2,065 (89%)	3,510 (88%)	
Non-regular worker	245 (11%)	489 (12%)	
How do you feel about your current financial situation?			<0.01
Very difficult	303 (13%)	262 (7%)	
Slightly difficult	1,149 (50%)	1,395 (35%)	
Fairly difficult	628 (27%)	1,483 (37%)	
Comfortable	230 (10%)	859 (21%)	
Self-rated health			<0.01
Very good	248 (11%)	223 (6%)	
Good	795 (34%)	589 (15%)	
Fair	866 (38%)	1,756 (44%)	
Bad	401 (17%)	1,431 (36%)	
Felt alone			<0.01
Never	1,696 (73%)	2,065 (52%)	
Usual	506 (22%)	1,417 (35%)	
Always	108 (5%)	517 (13%)	
Current smoker	517 (22%)	1,135 (28%)	<0.01
Alcohol consumption			<0.01
High frequency (4–7 days a week)	541 (23%)	1,085 (27%)	
Low frequency (1–3 days a week)	625 (27%)	1,161 (29%)	
Never	1,144 (50%)	1,753 (44%)	
Exercise			0.64
High frequency (4–7 days a week)	244 (11%)	396 (10%)	
Low frequency (1–3 days a week)	574 (25%)	1,020 (39%)	
Never	1,492 (65%)	2,583 (65%)	
Comorbidities, yes	1,137 (49%)	2,113 (53%)	<0.01

Abbreviations: JPY, Japanese yen.

Table 2 shows the ORs for the relationship between each SES variable and the lack of accommodations from companies. In the age- and sex-adjusted model, marriage and educational background were significantly associated with a lack of accommodation. Employment status also showed a weak association (OR = 1.17, 95% confidence interval (95% CI): 0.99–1.38, *p* = 0.06), indicating a marginal trend toward significance. In the fully adjusted models, none of these associations remained statistically significant. All other factors were associated with the lack of accommodation from companies in the multivariate analysis. The OR for non-desk workers compared with desk workers was 1.15 (95% CI: 1.04–1.28, *p* = 0.01). The lower the income, the higher the OR. Compared with the highest income group, the OR for the lowest income group was 1.66 (95% CI: 1.39–1.97, *p* < 0.01). The worse the respondents reported their subjective economic situation to be, the larger the OR. The OR for those who reported a difficult financial situation was 8.04 (95% CI: 4.73–14.10, *p* < 0.01) compared with those who reported a comfortable financial situation.

**Table 2:** Association between socioeconomic status and lack of receiving accommodation

	Model 1				Model 2				Model 3			
	OR	95% CI		*p*	OR	95% CI		*p*	OR	95% CI		*p*
Marital status												
Married	Reference				Reference				Reference			
Unmarried	1.35	1.14	1.62	<0.01	1.16	0.96	1.39	0.12	1.11	0.92	1.34	0.30
Divorced/bereavement	1.14	1.02	1.28	0.03	1.00	0.88	1.13	>0.9	0.99	0.88	1.12	0.90
Job type												
Mainly desk worker	Reference				Reference				Reference			
Non-desk worker	1.21	1.09	1.34	<0.01	1.15	1.04	1.28	0.01	1.15	1.04	1.28	0.01
Annual equivalent household income (JPY)												
9,000,000–	Reference				Reference				Reference			
7,000,000–8,990,000	0.99	0.84	1.17	>0.9	0.99	0.83	1.17	0.90	0.99	0.84	1.18	>0.9
5,000,000–6,990,000	1.29	1.10	1.51	<0.01	1.27	1.08	1.49	<0.01	1.29	1.10	1.52	<0.01
3,000,000–4,990,000	1.47	1.26	1.71	<0.01	1.44	1.23	1.69	<0.01	1.47	1.26	1.72	<0.01
-2,990,000	1.70	1.43	2.02	<0.01	1.66	1.39	1.97	<0.01	1.68	1.40	2.00	<0.01
Education background												
Vocational school/college, university, graduate school	Reference				Reference				Reference			
High school	1.16	1.03	1.31	0.02	1.08	0.96	1.22	0.20	1.04	0.92	1.18	0.50
Junior high school	1.64	1.05	2.62	0.03	1.46	0.94	2.34	0.10	1.33	0.85	2.15	0.20
Employment status												
Regular worker	Reference				Reference				Reference			
Non-regular worker	1.17	0.99	1.38	0.06	1.01	0.86	1.20	0.90	1.03	0.87	1.22	0.70
How do you feel about your current financial situation?												
Very comfortable	Reference				Reference				Reference			
Comfortable	2.05	1.20	3.61	0.01	2.09	1.22	3.67	0.01	2.17	1.27	3.82	0.01
Fairly difficult	2.66	1.60	4.58	<0.01	2.68	1.61	4.62	<0.01	2.74	1.64	4.74	<0.01
Slightly difficult	5.17	3.10	8.91	<0.01	5.16	3.08	8.93	<0.01	5.15	3.07	8.92	<0.01
Very difficult	8.09	4.79	14.10	<0.01	8.04	4.73	14.10	<0.01	7.85	4.60	13.8	<0.01
Number of employees												
1000–	Reference				Reference				Reference			
500–999	1.23	1.01	1.50	0.04	1.21	1.00	1.48	0.05	1.20	0.99	1.47	0.07
100∼499	1.46	1.26	1.69	<0.01	1.40	1.21	1.63	<0.01	1.39	1.20	1.62	<0.01
30∼99	1.32	1.12	1.54	<0.01	1.23	1.05	1.44	0.01	1.24	1.06	1.45	0.01
-29	1.33	1.15	1.53	<0.01	1.21	1.05	1.40	0.01	1.21	1.05	1.40	0.01
Support from supervisor												
Extremely	Reference				Reference				Reference			
Very much	1.72	1.36	2.19	<0.01	1.74	1.37	2.22	<0.01	1.79	1.41	2.29	<0.01
Somewhat	4.28	3.38	5.46	<0.01	4.27	3.37	5.45	<0.01	4.38	3.44	5.59	<0.01
Not at all	13.10	9.95	17.3	<0.01	12.90	9.81	17.1	<0.01	12.9	9.79	17.1	<0.01
Support from coworker												
Extremely	Reference				Reference				Reference			
Very much	1.40	1.11	1.77	<0.01	1.40	1.10	1.77	<0.01	1.42	1.12	1.81	<0.01
Somewhat	2.98	2.36	3.77	<0.01	2.93	2.31	3.71	<0.01	2.96	2.34	3.76	<0.01
Not at all	8.16	6.12	10.9	<0.01	7.89	5.90	10.6	<0.01	7.77	5.81	10.5	<0.01

Model 1 was adjusted for age and sex.

Model 2 was adjusted for sex, age, equivalent household income, and educational background.

Model 3 was adjusted for sex, age, equivalent household income, educational background, alcohol, smoking, exercise habits, and comorbidities.

Abbreviations: CI, confidence interval; JPY, Japanese Yen; OR, odds ratio.

## 4 Discussion

This cross-sectional study of workers with health problems during the COVID-19 pandemic showed a relationship between SES and the lack of accommodation for their needs by companies. More specifically, non-desk workers, workers with low equivalent household incomes, workers with low subjective economic status, workers in companies with a small workforce, and workers who felt unsupported by their supervisors and coworkers were significantly more likely to report not receiving job accommodation from their companies.

The present finding that the needs of workers of vulnerable SES with health problems were not accommodated by their companies is consistent with the previous literature. Among 4,937 workers with functional limitations or disabilities in the U.S., men did not receive accommodations, whereas college graduates, older workers, and full-time workers did [25]. In a survey of 1,514 cancer survivor workers in France, workplace accommodation was positively associated with female gender, large company size, and full-time contracts at diagnosis [19]. A study of 267 breast cancer patients in the U.S. reported that those with low incomes did not receive workplace accommodations [37]. A study of business associations in the U.S. noted that small businesses were significantly less likely than larger employers to make accommodations for employees with disabilities [38].

In this study, “job accommodation” was operationally defined as specific workplace adjustments or modifications aimed at enabling workers with health problems or disabilities to perform their jobs effectively. This does not include general welfare benefits or provisions for the broader workforce. Based on this definition, there are several possible reasons why workers of vulnerable SES with health problems have difficulty receiving accommodations from companies. First, companies need well-organized systems and occupational health services to care for workers with health problems. Such systems are generally more extensive in large companies and those with better management conditions [38,39]. They also have systems to facilitate accommodation of workers’ needs, partly because of social responsibility and compliance requirements.

Second, the ease of accommodation differs depending on the nature of the work. For example, employees with desk work duties can be more easily supported than those with non-desk work duties. While working hours can be reduced and hospital visits taken into account for desk work tasks, non-desk work tasks require changes in the nature of the work itself, and such changes may be complicated.

Third, previous studies have shown that a worker’s employment stability affects the ease with which the worker can apply to receive accommodations. Workers with employment and financial stability, and those who perform specialized work, are more likely to receive accommodations from their company [19,25,26]. Conversely, workers with unstable financial and employment statuses have reported that health problems discourage them from continuing to work [26,27]. However, our study found that while employment status initially showed an association with job accommodation, this association disappeared after adjusting for SES and lifestyle factors. This suggests that factors like income level, workplace environment, and social support may have a greater impact on receiving accommodations than employment status alone. While prior studies indicate that non-regular workers are disadvantaged [19,25,27], our findings suggest that employment type is not the sole determinant, highlighting the need for broader workplace policies to support all workers with health issues.

Fourth, there is an association between being in a supportive relationship and a worker’s likeliness of applying for workplace accommodations. In this study, married workers reported receiving more accommodation than unmarried workers. Additionally, we found that workers who felt supported by their supervisors and coworkers received more accommodations than those who felt unsupported. Those who have such supportive relationships could possibly encourage workers to request workplace accommodation or at least feel more comfortable applying for it.

Conversely, SES may be negatively affected by the lack of accommodations from companies for workers with poor health. If workers with poor health do not receive accommodations, they may work fewer hours, do fewer jobs, or be less able to perform their duties. As a result, workers’ SES worsens as they become unemployed or move to jobs with lower wages and/or poorer working conditions.

Our results suggest that workers with health problems and disadvantaged SESrequire further public support additional to accommodations by companies. Workers with health problems are known to have a high risk of leaving their jobs [40]. Vulnerable SES is also associated with a high risk of turnover, and workers with both health problems and low SES thereby suffer a double burden. Some companies are making efforts to ensure that employees with health problems can continue working. However, for workers with unstable SESwho cannot receive such support, the company’s efforts are insufficient to cover the costs. In addition, while companies are motivated to prevent job turnover, support for those who have left the workforce is not the company’s responsibility. Health-impaired workers with disadvantageous SES are at high risk of falling into poverty. Such downward movement to the lower strata of society is itself a risk for society [41]. To avert this situation eventually leading to social security problems, workers must receive appropriate support and accommodations not only through the efforts of the company but also through the social system.

This study has several limitations. First, we did not identify the specific nature of health problems. Health problems include a wide range of illnesses, from mental illness to physical illness, and each requires different accommodations. The association between SES and accommodation differs from disease to disease. Second, we did not identify what specific accommodations workers required. There is a wide range of types of accommodations, including physical work environment adjustments, changes in the job description, obtaining treatment opportunities, etc. Depending on the SES, some types of accommodation are easier to receive than others. For example, desk workers and manual laborers receive different types of accommodation. Third, there are other indicators within SES, such as occupational categories, but these were not examined in this study. Additionally, the inherent flexibility of certain jobs held by highly educated, senior, or full-time workers could influence the ability to provide job accommodations. However, this study specifically focuses on explicit workplace adjustments for workers with health problems, distinct from general job flexibility or privileges associated with SES. Fourth, the validity of the single self-reported question to assess the situation regarding accommodation requirements is undetermined. There may be situations where workers are unaware of accommodations already in place, or where they feel unable to express their needs to their companies. Fifth, our study population did not include those who had already become unable to work due to not receiving accommodations, or those who had resigned before accommodation was offered due to the severity of their disease. It was previously reported that some cancer patients who did not receive accommodations quit their job [25]. Sixth, the sample size for this study is relatively small, which limits the generalizability of the findings. Seventh, the survey was conducted during a pandemic, which may have affected the results. Further research is needed to determine if associations such as those identified here will be found after the pandemic.

In conclusion, this study found a relationship between vulnerable SES and accommodations by their companies for workers with health problems, among a large group of workers across Japan. The lack of accommodations for vulnerable SES workers with health problems may further worsen their SES and health conditions. Our findings underline the importance of providing job accommodation for health-impaired workers with vulnerable SES to reduce social disparities.

## CRediT authorship contribution statement

**Yu Igarashi:** Writing – original draft. **Seiichiro Tateishi:** Writing – review & editing, Investigation, Funding acquisition. **Arisa Harada:** Writing – review & editing, Validation. **Kosuke Mafune:** Writing – review & editing, Validation, Funding acquisition. **Mayumi Tsuji:** Writing – review & editing, Validation, Funding acquisition. **Akira Ogami:** Validation, Methodology, Investigation, Funding acquisition. **Tomohisa Nagata:** Writing – review & editing, Validation, Funding acquisition. **Ryutaro Matsugaki:** Writing – review & editing, Validation, Funding acquisition. **Yoshihisa Fujino:** Writing – review & editing, Writing – original draft, Validation, Project administration, Methodology, Investigation, Funding acquisition, Data curation.

## Funding

This study was supported and partly funded by a research grant from the University of Occupational and Environmental Health, Japan (no grant number); Japanese Ministry of Health, Labour and Welfare (H30-josei-ippan-002, H30-roudou-ippan-007, 19JA1004, 20JA1006, 210301-1, and 20HB1004); Anshin Zaidan (no grant number), the Collabo-Health Study Group (no grant number) and Hitachi Systems, Ltd. (no grant number), and scholarship donations from Chugai Pharmaceutical Co., Ltd. (no grant number). No funder was involved in the study design, collection, analysis, interpretation of data, the writing of this article or the decision to submit it for publication.

## Conflicts of interest

All authors declare no conflicts of interest associated with this manuscript.
